# IGF-1 Via PI3K/Akt/S6K Signaling Pathway Protects DRG Neurons with High Glucose-induced Toxicity

**DOI:** 10.1515/biol-2019-0056

**Published:** 2019-12-31

**Authors:** Chunhong Liu, Siyan Liu, Sheng Wang, Yi Sun, Xin Lu, Hao Li, Guibao Li

**Affiliations:** 1 Department of Anatomy, Shandong University School of Basic Medical Sciences, Jinan, 250012, China; 2 Department of Rheumatology, Shandong University Qilu Hospital, Jinan 250012, China; 3 Department of Rheumatology, Yantai Affiliated Hospital of Binzhou Medical University, Yantai 264100, China; 4 Department of Orthopaedics, Shandong University Qilu Hospital, Jinan 250012, China

**Keywords:** high glucose, neurotoxicity, dorsal root ganglion, insulin-like growth factor-1, activating transcription factor 3

## Abstract

Hyperglycemia-induced toxicity of neurons contributes to the pathogenesis and progression of diabetic neuropathy (DNP). High concentration glucose triggered reactive oxygen species (ROS) overproduction and induced cell apoptosis of neurons from dorsal root ganglion (DRG) in vitro. Currently, there is no effective therapeutic method to retard this devastating complication or neurotoxicity induced by high glucose. Insulin-like growth factor-1 (IGF-1) has multi-neurotrophic actions which need to be explored regarding its actions and mechanisms on relieving high glucose induced neurotoxicity. Herein, high concentration glucose was exposed to the DRG neurons in vitro. The effects of IGF-1 on relieving high glucose-induced neurotoxicity were evaluated. We illustrated that IGF-1 enhanced regeneration of neurites sent from DRG neuronal cell bodies and increased neuronal viability which inhibited by high glucose challenge. IGF-1 alleviated neuronal apoptosis caused by high glucose exposure. IGF-1 also suppressed the intracellular ROS overproduction and ATF3 expression upregulation which was induced by high glucose insult. The anti-neurotoxic effects of IGF-1 might be through restoration of prosurvival PI3K/Akt/S6K signaling. These data shed some light on the treatment of intractable DNP and suggested that IGF-1 might be a potential effective agent on relieving high glucose induced neurotoxicity.

## 1 Introduction

The devastating complication of diabetic neuropathy (DNP) in diabetic patients is one of the most common forms of diabetic complications [1]. The majority of patients with DNP suffer the distal symmetrical polyneuropathy (DSPN), a slowly progressive sensory predominant neuropathy, and experience numbness, tingling, pain, and weakness that typically starts in the toes and progresses up the feet, then the legs, which results from a symmetrical length-dependent dysfunction and damage of peripheral nerves [2]. It is generally agreed that hyperglycemia-induced toxicity of neurons contributes to the pathogenesis and progression of DNP, and high concentration glucose triggered reactive oxygen species (ROS) overproduction and induced cell apoptosis of neurons from dorsal root ganglion (DRG) in vitro [3]. Current management of DNP rests on three tenets: intensive glycemic control; pathogenetic therapies; and symptomatic treatment [4], which possess insufficient efficacy. Accordingly, there is urgent need for us to clarify the underlying mechanism of DNP and develop better therapies to treat this refractory condition.

The extensively distributed insulin-like growth factor-1 (IGF-1) in various tissues possesses powerful growth-promoting [5] and protective effects on neurons [6]. Moreover, dysregulation of this neural trophic factor is demonstrated to have pathogenic relevance with DNP [7]. Hence, modulation of IGF-1 level and its downstream signaling pathways might be a potential candidate to treat DNP, and the mechanism as well as the efficacy of this therapy requires further exploration.

Activating transcription factor 3 (ATF3) is considered as a reliable indicator of neuronal damage [8] and could be induced by a diverse array of noxious stimuli in DRG neurons [9,10]. Additionally, recent studies indicate the involvement of ATF3 upregulation in the genesis and maintenance of neuropathic pain in a wide range of pathological models [11, 12, 13]. However, the role of ATF3 expression in DNP remains unclear.

Herein, high concentration glucose was exposed to the DRG neurons in vitro. The high glucose challenge induced neurotoxicity which was reversed by IGF-1 was evaluated. We illustrated that IGF-1 enhanced regeneration of neurites sent from DRG neuronal cell bodies and increased neuronal viability which was inhibited by high glucose challenge. IGF-1 also alleviated neuronal apoptosis, inhibited intracellular ROS production, and downregulated ATF3 expression in the presence of high concentration glucose. The anti-neurotoxic effects of IGF-1 might be through restoration of prosurvival phosphatidyl inositol 3-kinase (PI3K)/Akt/S6K signaling. These data shed some light on the treatment of intractable DNP and suggested that IGF-1 might be a potential effective agent on relieving high glucose induced neurotoxicity.

## 2 Experimental Procedures

### 2.1 Culture of neurons from DRG

The DRG was removed from newborn Wistar rats under anesthesia. The preparation of the culture used in this study was referred as a previous report [14]. Briefly, DRG tissue explants from each newborn rat were processed by digestion with trypsin (0.25%), centrifugation (1×10^3^ rpm, 5 minutes), resuspension and homogenisation with a glass pipette, and filtration through a 130 μm filter. According to the experimental research aims, the cells were cultured in different clusters with different concentrations of the cells. The 24-well clusters were used for total neurite length measurement of single neurons with the cell density plating at 5 ×10^4^ cells/well and for double fluorescence labeling with the cell density plating at 1 ×10^5^ cells/well. The 24-well clusters were used for Western blot and polymerase chain reaction (PCR) with the cell density plating at 5 ×10^5^ cells/ well. The 96-well clusters were used for neuronal viability with the cell density plating at 5,000 cells in each well. These plated cells, both in 24-well and 96-well clusters, were incubated with 5% CO_2_ at 37°C for the different incubation stage as the following designed stimulation procedures. For the first 24 hours, cells were only cultured in medium (DMEM/F-12 with 5% fetal bovine serum and 20 μl/ml 1×B-27) to allow neuronal cell attachment and neurite outgrowth. In the second 24 hours, the cells were cultured in medium supplemented with cytosine arabinoside (ara-C, 5 μg/ml). This is an incubation stage for inhibiting non-neuronal cell growth by ara- C. In the third 24 hours, the cells were incubated in different agent stimulated conditions to complete the final experiment. The stimulating conditions were described in the section 2.2.

**Ethical approval**: The research related to animals use has been complied with the National Institute of Health Guide for the Care and Use of Laboratory Animals (eighth edition, 2010) and approved the Animal Experimentation Ethical Committee in Shandong University.

### 2.2 Treatment with stimulating agents at experimental stage

Stimulating agents were added in the following randomly divided groups. (1) High glucose group: The stimulating agent was 45 mmol/ L glucose for the final concentration in medium. The basic culture medium contains 25 mmol/ L glucose. The high glucose group was supplemented with an additional 20 mmol/ L glucose making the final glucose concentration 45 mmol/ L. (2) High glucose + IGF-1 group: The stimulating agents were 45 mmol/ L glucose plus 20 nmol/L IGF-1 in medium. (3) High glucose + IGF-1 + LY294002 (a P13K inhibitor) group: The stimulating procedure was 10 μmol/L LY294002 incubation for 30 minutes and following 45 mmol/ L glucose plus 20 nmol/ L IGF-1 in the medium. In this group, pre-incubation with LY294002 aimed to block PI3K and its downstream signaling. (4) Mannitol group: The stimulating agent was only 20 mmol/L mannitol. The basic culture medium contains 25 mmol/L glucose. The mannitol group was supplemented with 20 mmol/ L mannitol to simulate the same hyperosmotic state. (5) Control: No stimulating agent was added. All above experimental conditions were maintained for 24 hours.

### 2.3 Neurite length measurement

The neurite length of each neuron may represent the regenerative state of a neuron in culture condition. In this experiment, the neurites were firstly labeled with βIII-tubulin fluorescent staining and secondly analyzed by using ImageJ software as a previous study [15]. Briefly, the cells were washed with phosphate buffer saline (PBS, 0.1 mol/ L). This was followed by fixation with cold (4°C) paraformaldehyde (4%, pH 7.4, 20 minutes). Blockade of non-specific sites and permeabilization of the cells were carried out with goat serum (2%) in Triton (0.3%) PBS. Incubation with the first antibody (1:1000 mouse monoclonal anti-βIII-tubulin) was at 4°C cold room for 16-18 hours. Incubation with the second antibody (1:100 goat anti-mouse conjugated to Cy2) was conducted in a dark chamber for 45 minutes. The cells were mounted with anti-fade fluorescence mounting medium. The length of all the neurites in each neuron was measured using ImageJ software.

### 2.4 Neuronal viability assay

Neuronal viability was analyzed with CCK-8 kit (Beyotime, Shanghai, China) containing highly water-soluble tetrazolium salt. The measurement procedure was according to the previous studies for this specific cell counting kit [14,15]. Briefly, using 96-well culture plate, 10 μl of the agent was added to each well, and the intensity was assessed 4 hours later with a Micoplate Photometer (Multiscan MK3, Thermo Labsystems, Waltham, MA) at 450 nm wavelength. The neuronal viability in control group was taken as the standard. The neuronal viability in other experimental groups was obtained from the comparison with the control standard.

### 2.5 Observation of neuronal apoptosis

Neuronal apoptosis was indicated by Hoechst 33342 fluorescence staining and caspase3 protein expression levels. The staining procedure for Hoechst 33342 was similar to the previous studies [14, 15, 16, 17]. Briefly, fixation of the cells was carried out with paraformaldehyde (4%, pH 7.4, 10 minutes) incubation. A concentration of 10 μg/ml Hoechst 33342 (37°C, 15 minutes) was applied for fluorescence staining. A fluorescent microscope (IX-70, Olympus) was used for assessment of Hoechst 33342 fluorescence excited by UV wavelengths (340 nm). The apoptotic neurons would have fragmented or condensed chromatin or shrunken nuclei. By comparing the number of apoptotic total neurons, the percentage of the apoptotic neurons could be calculated. A Western blot assay for caspase3 protein expression was also used as an indicator of neuronal apoptosis. The Western blot analysis procedure is described in Section 2.8.

### 2.6 Detection for ROS levels

2′,7′-dichlorodihydrofluorescein diacetate (DCFH-DA) (Sigma, St. Louis, MO), a cell-permeable oxidation-sensitive fluorescent probe, was used for monitoring intracellular ROS production. The measurement procedure for ROS level in DRG neurons in culture was similar to the previous studies [15,16]. Briefly, a final concentration of 10 μmol/L DCFH-DA was incubated (37°C, 30 minutes) in the culture medium. After that, the unbound fluorescent probe was removed with PBS. A fluorescent microscope was used for taking microphotograph with 485 nm excitation and 530 nm emission. ImageJ software was used for analyzing the intensity of fluorescence which represents the relative amount of ROS levels.

### 2.7 Real time-PCR for detecting ATF3 mRNA

ATF3 mRNA expression of DRG neurons with different stimulating agents was detected with a real-time PCR technique. Glyceraldehyde 3-phosphate dehydrogenase (GAPDH) mRNA level was used for internal control. TRIzol (TakaRa Biotechnology) was used for isolating total RNA from DRG cells under different stimulating conditions. A cDNA synthesis kit (Thermo Scientific Molecular Biology) was used for cDNA synthesis. The sequences of the synthetic oligonucleotide primers are listed in table 1. Quantitative PCR was carried out with SYBR Green dye (Thermo Scientific Molecular Biology) and amplification was performed with the synthetic oligonucleotide primers. The PCR reaction was undertaken at 50°C for 2 minutes, 94°C for 15 minutes, followed by 40 cycles at 94°C for 15 seconds, 58°C for 30 seconds, and 72°C for 30 seconds. The results of PCR analysis were presented with 2^-ΔΔCt^ method [18].

**Table 1:** The sequences of oligonucleotide primers

Genes	Primer sequences
ATF3	5’-CCT GCA GAA GGA GTC AGA GAA-3’ (coding sense)
	5’-CGT TCT GAG CCC GGA CGA TA-3’ (coding antisense)
GAPDH	5’-GGC ACA GTC AAG GCT GAG AAT G-3’ (coding sense)
	5’-ATG GTG GTG AAG ACG CCA GTA-3’ (coding antisense)

### 2.8 Western blot for detecting ATF3, caspase3, pAkt, and pS6K proteins

ATF3 and caspase3 protein expression of DRG neurons after different stimulation for 24 hours was detected with the Western blot technique. The pAkt, Akt, S6K, and pS6K expression of DRG neurons after different stimulating for 30 minutes was also detected with Western blot.

The b-actin protein expression was used as an internal control. Fresh DRG cells were lysed in RIPA buffer (Beyotime Biotechnology) which contained protease and phosphatase inhibitors (Roche) on ice for 20 minutes. The dissolved DRG tissue was centrifuged (10,000 g, 20 minutes) for supernatant collection. The protein samples (50 μg) were loaded into each lane and total protein was separated in 12% SDS gel and transferred to nitrocellulose membrane which was followed by a blocking step with 5% nonfat milk at room temperature for 2 hours. After that, the samples were incubated with each prepared primary antibody (4°C, overnight) and the following corresponding secondary antibody at room temperature for 2 hours. After finishing the immunoreaction, the captured images were quantitatively analyzed using ImageJ software. The antibodies used in this experiment were listed in table 2.

**Table 2:** The antibodies for immunoblotting

category	antibodies	concentration	source
primary	mouse anti-ATF3 monoclonal IgG	1:1000	Abcam, Cambridge, MA
primary	rabbit anti-caspase3 monoclonal IgG	1:1000	Cell Signaling Technology, Danvers, MA
primary	rabbit anti-pAkt monoclonal IgG	1:1000	Cell Signaling Technology, Danvers, MA
primary	rabbit anti-Akt monoclonal IgG	1:1000	Cell Signaling Technology, Danvers, MA
primary	rabbit anti-pS6K monoclonal IgG	1:1000	Cell Signaling Technology, Danvers, MA
primary	rabbit anti-S6K monoclonal IgG	1:1000	Cell Signaling Technology, Danvers, MA
primary	mouse anti-β-actin monoclonal IgG	1:1000	Santa Cruz Biotechnology, Santa Cruz, CA
secondary	goat anti-rabbit IgG-HRP	1:6000	Beijing Sequoia Jinqiao Biological Technology Co., Ltd., Beijing, China
secondary	goat anti-mouse IgG-HRP	1:3000	Beijing Sequoia Jinqiao Biological Technology Co., Ltd., Beijing, China

### 2.9 Double fluorescence staining for determining the proportion of ATF3-expressing neurons

Microtubule-associated protein 2 (MAP2) was used for staining all subtypes of DRG neurons. ATF3-expressing neurons were also stained in the same group of the DRG neurons with a different color to distinguishing them from the ATF3-negative neurons but positive to MAP2. The double fluorescence staining protocol was similar to the previous studies [14,15]. Briefly, after quickly washing with PBS for cleaning the cells, fixation with cold (4°C) paraformaldehyde (4%, pH 7.4, 20 minutes) was followed. Blockade of non-specific sites and cell permeabilization were carried out with normal goat serum (2%) in 0.3% Triton PBS. Incubation with the primary antibodies was at 4°C cold room for 16-18 hours. Incubation with the corresponding secondary antibodies was in a dark chamber for 45 minutes. The samples were mounted with anti-fade fluorescence mounting medium. Cell counting for MAP2-positive neurons (total neurons) and ATF3-expressing neurons was done for calculating the proportion of ATF3-positive neurons.

### 2.10 Statistical Analysis

Mean ± SD was used for reporting the quantitative data obtained in this study. SPSS (version 17.0) was used for the statistical analysis. A non-parametric test was used for analyzing abnormal distributed data. The normally distributed data were assessed with one way analysis of variance followed by Student-Newman-Keuls test (homogeneityof variance) or Dunnett’s T3 test (heterogeneity of variance). A *P* value < 0.05 was taken as significant.

## 3 Results

### 3.1 IGF-1 restores PI3K/Akt/S6K signaling impaired by high glucose stimuli

**Figure 1:**
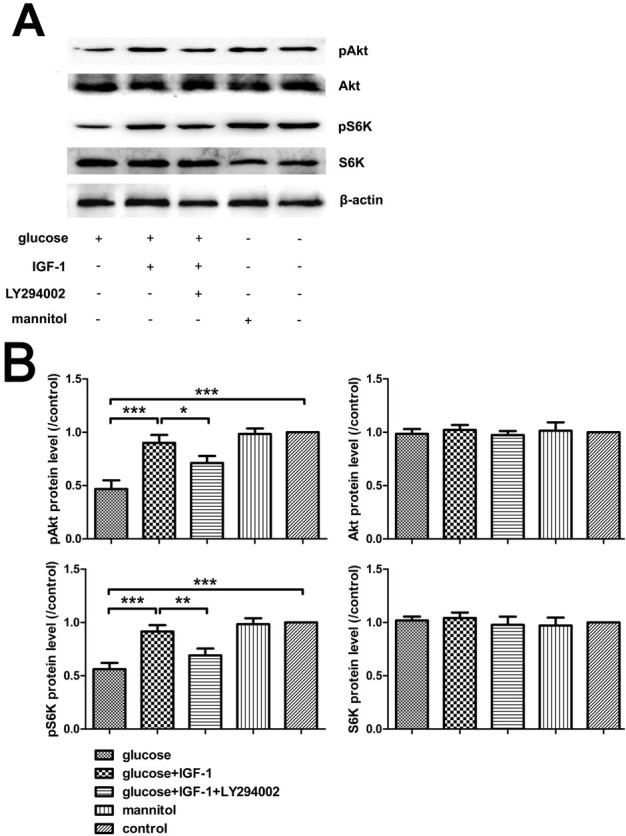
**Phosphorylation of Akt and S6K analysis with Western blot**. (A) Immunoreactive bands. (B) Analysis for pAkt, Akt, pS6K, and S6K protein levels. Mean ± SD (n = 5). *P<0.05, **P<0.01, ***P<0.001.

DRG neurons were harvested after stimulation with the agents used as described in Section 2.2 for 30 minutes, and then the levels of pAkt and pS6K were assessed by Western blot tests. The results showed that high glucose exposure decreased the phosphorylation level of Akt and S6K. IGF-1 incubation rescued Akt and S6K activation in the presence of high glucose. The effect was inhibited by pretreatment with LY294002, a PI3K inhibitor. According to the effective inhibitory actions of LY294002, exogenous IGF-1 could restore the PI3K/Akt/S6K signaling impaired by high glucose insult, which might be a mechanism underlying the neuroprotective effect of IGF-1 (Figure 1).

### 3.2 IGF-1 reverses neurite shortening after high glucose stimulation

**Figure 2:**
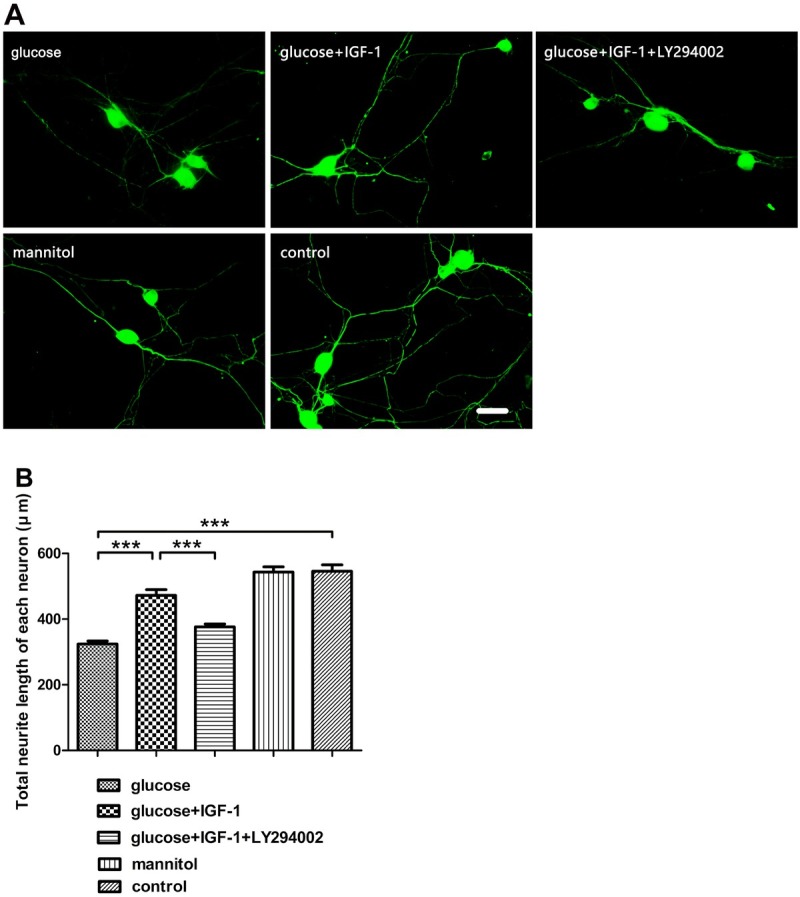
**Neurite length measurement**. (A) βIII-tubulin fluorescence labeling for neurites. (B) Analysis for neurite length. Scale bar = 50 μm. Mean ± SD (n = 5). ***P<0.001.

High glucose stimulation can cause neurite retraction. The length of neurite extended from each neuron was measured after each stimulation procedure finished to investigate the protective effects of exogenous IGF-1. The results showed that exogenous IGF-1 could reverse neurite shortening after high glucose stimulation. The effect of IGF-1 on neurite elongation might be through activation of PI3K/Akt/S6K signaling. Mannitol did not affect the length of neurite, suggesting that osmotic pressure did not influence neurite outgrowth and that neurite loss was caused by high glucose stimulation (Figure 2).

### 3.3 IGF-1 protects DRG neurons against high glucose-induced apoptosis and high glucose-reduced cell viability

**Figure 3:**
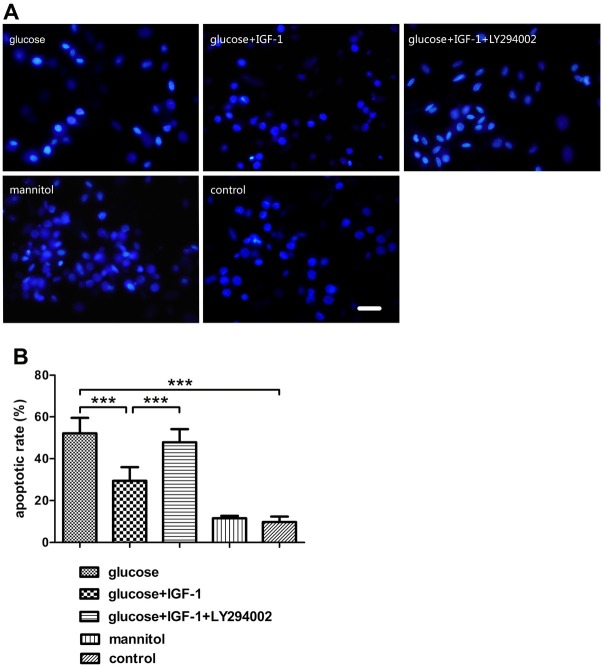
**Determination of the neuronal apoptotic rate**. (A) Hoechst 33342 staining for DRG neurons. (B) Analysis for the apoptotic rate of DRG neurons. Scale bar = 50 μm. Mean ± SD (n = 5). ***P<0.001.

**Figure 4:**
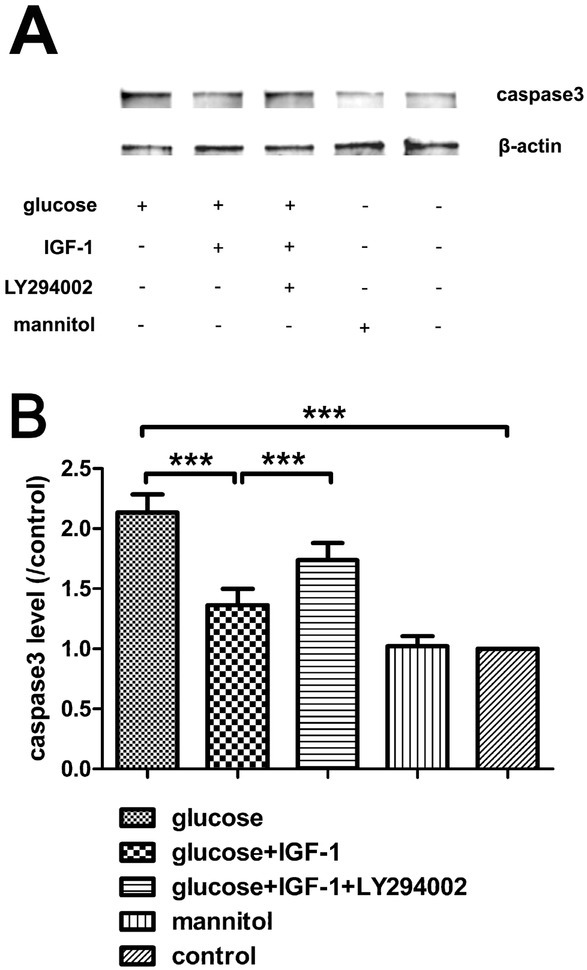
**Western blot analysis for caspase3 protein expression**. (A) Immunoreactive bands. (B) Analysis for caspase3 protein levels. Mean ± SD (n = 5). ***P<0.001.

**Figure 5:**
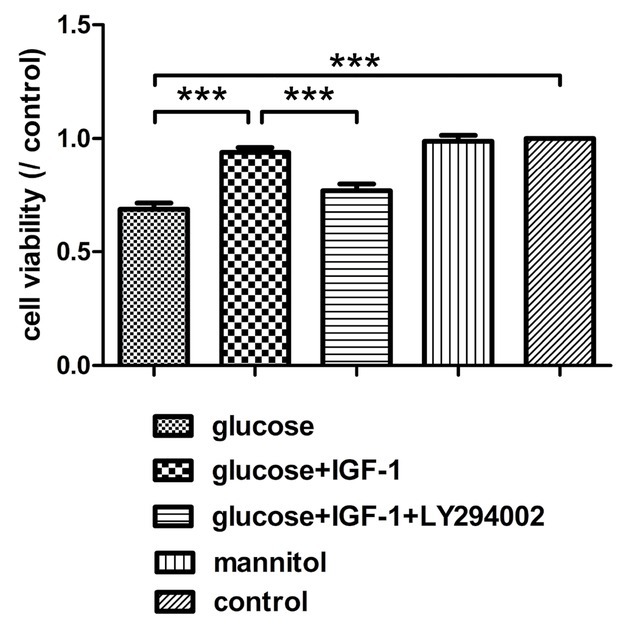
**Analysis for cell viability of DRG neurons**. Scale bar = 50 μm. Mean ± SD (n = 5). ***P<0.001.

In this study, after treating cultured DRG neurons with different agents for 24 hours, the protective effects of IGF-1 against high glucose-induced apoptosis were assessed by Hoechst 33342 staining and caspase3 expression levels. High glucose insult increased the percentage of apoptotic DRG neurons and caspase3 expression levels. IGF-1 could block these effects and protect neurons from high-glucose-fomented apoptosis (Figure 3 and 4). The cell viability was assessed by CCK8 assay, cell viability was reduced with high glucose stimuli. The inhibitory actions of high glucose on cell viability could be decreased by exogenous IGF-1 incubation (Figure 5). The beneficial effects of exogenous IGF-1 against high glucose exposure on reducing apoptosis and increasing cell viability could be blocked by using a PI3K inhibitor LY294002. Mannitol treatment did not have significant effects on neuronal apoptosis and viability.

### 3.4 IGF-1 decreased intracellular ROS levels induced by high glucose treatment

**Figure 6:**
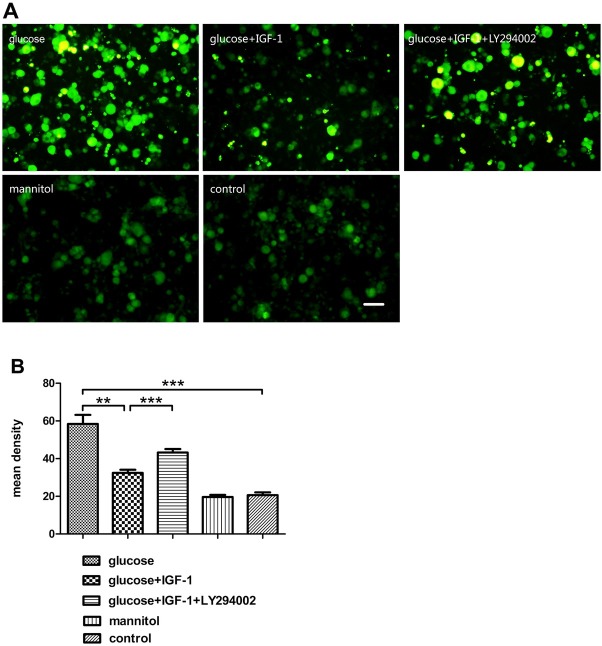
**Intracellular ROS production determination in DRG neurons**. (A) Intracellular ROS labeled by DCFH-DA. (B) Analysis for fluorescent density. Scale bar = 50 μm. Mean ± SD (n = 5). **P<0.01, ***P<0.001.

Neurons are vulnerable to oxidative stress characterized by elevated ROS level. In this study, after culturing for 48 hours, DRG neurons were incubated under different experimental conditions for another 24 hours followed by measurement of ROS production in neuronal cells. The amount of ROS production in neurons was reflected by measurement of the fluorescence intensity as determined with DCFH-DA fluorescence probes in this experiment. High glucose stimulation elevated intracellular ROS production and high glucose-induced ROS elevation could be inhibited by exogenous IGF-1. Pretreatment with LY294002 blocked the effect of IGF-1 on ROS production inhibition. High osmotic pressure induced by mannitol did not change the intracellular ROS production level as compared with ROS production level in neurons from the control group (Figure 6).

### 3.5 IGF-1 counteracts high glucose induced ATF3 mRNA and protein expression

**Figure 7:**
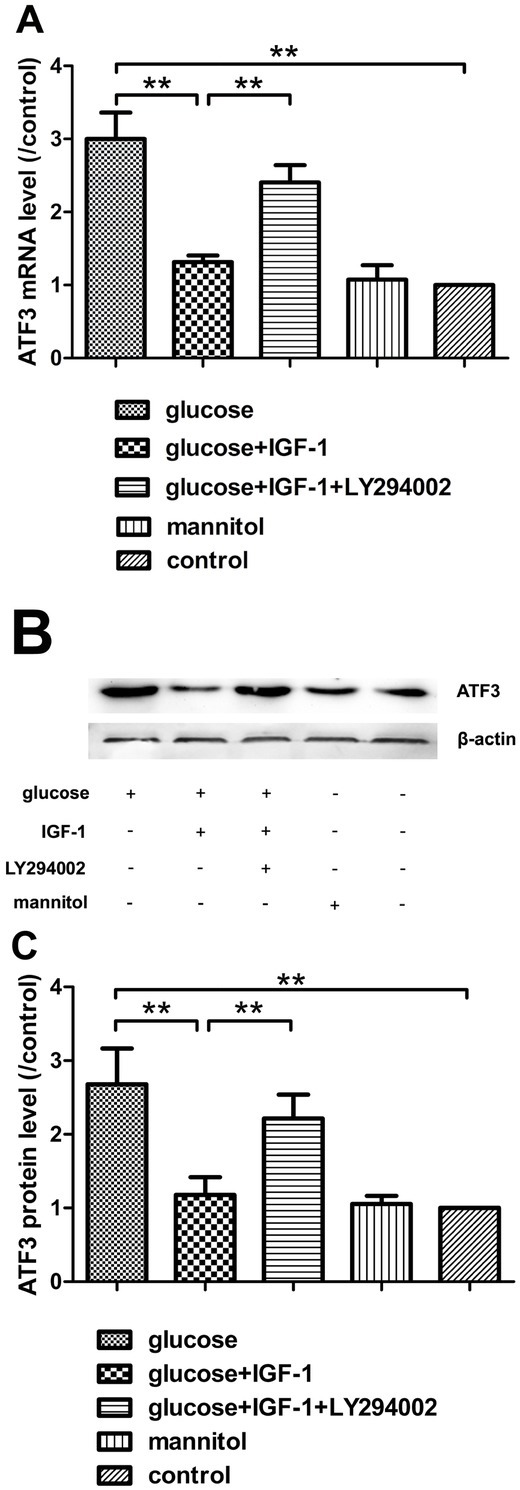
**The mRNA and protein levels of ATF3**. (A) Analysis for ATF3 mRNA levels. (B) ATF3 protein immunoblotting bands. (C) Analysis for ATF3 protein levels. Mean ± SD (n = 5). **P<0.01.

Here, the alterations of ATF3 mRNA and protein levels were measured after different stimulation conditions for 24 hours. The results illustrated that, upon high glucose stimulation for 24 hours, ATF3 expression increased both in mRNA and protein levels. Incubation with exogenous IGF-1 could significantly down regulate ATF3 expression which was elevated by high glucose insult. The actions of IGF-1 on ATF3 expression could be inhibited by LY294002 preincubation suggesting this downstream signaling involved in the actions of IGF-1 on ATF3 expression at the stress status induced by high glucose challenge. ATF3 expression was not significantly affected in mannitol group, suggesting that high osmotic pressure had little influence on the expression of this transcription factor in neurons (Figure 7).

### 3.6 IGF-1 antagonizes the effect of high glucose on ATF3 expression in situ

**Figure 8:**
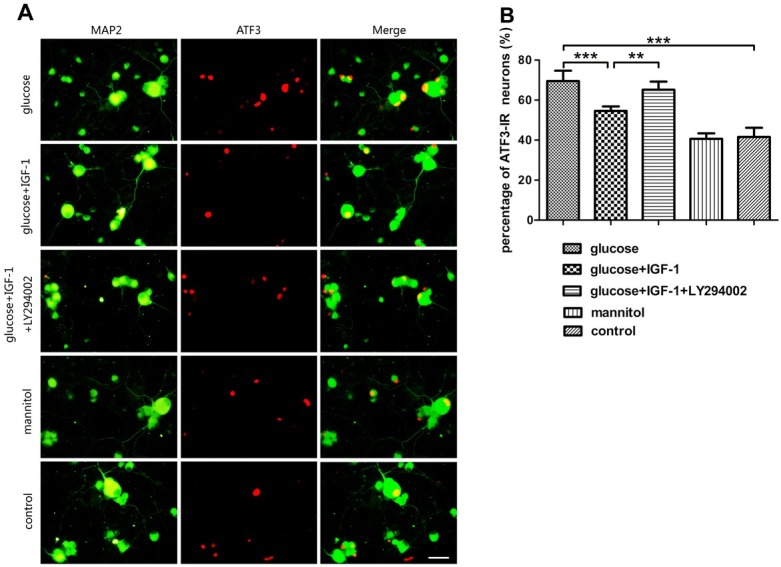
**Analysis for ATF3 expression in situ in DRG neuron**. (A) MAP2 and ATF3 double fluorescence staining. (B) The percentage of ATF3-immunoreactive (IR) neurons. Scale bar = 50 μm. Mean ± SD (n = 5). **P<0.01, ***P<0.001.

In the previous section, results showed that IGF-1 counteracted the effects of high glucose exposure on ATF3 mRNA and protein expression, which prompted us to further explore the alteration of in situ expression of this transcription factor by using a double fluorescence staining technique with MAP2 and ATF3. All the DRG neurons in the culture can be detected by MAP2 immunoreactions. By checking the simultaneous immunoreactivity for ATF3 in a group of subsets in all the MAP2-marked neurons, the proportion of ATF3-exprressing neurons can be obtained. In parallel with the ATF3 mRNA and protein levels, high glucose treatment significantly elevated the proportion of ATF3-exprressing neurons. These results imply that a high glucose challenge not only increased the amount of ATF3 protein, but also increased the number of DRG neurons which express ATF3. The elevated proportion of ATF3-expressing neurons induced by high glucose treatment was significantly inhibited by application of exogenous IGF-1. The effect produced by IGF-1 on ATF3-positive neuronal proportion could be suppressed by LY294002 pretreatment suggesting the PI3K/Akt/S6K signaling activation was involved in mediating IGF-1 induced actions. Mannitol treatment did not affect ATF3 expression in situ (Figure 8).

## 4 Discussion

IGF-1, coded by the *igf1* gene, is a pluripotent cytokine exerting its effects in virtually all cell types, and plays a vital role in the nervous system, it has been reported that IGF-1 promotes survival of neurons [19], neurogenesis and proliferation [20], outgrowth of axons [5], regeneration of axons [21], maintenance of synaptic connections [22], and neuronal functions in many aspects [23]. However, at the onset stage of DNP, this cytokine and its signaling pathways are dysregulated, which might be closely related to the pathogenesis and progression of DNP. Previous reports have demonstrated that diabetic patients with DNP have lower IGF-1 levels in serum [24,25]. Moreover, sensory deficit similar to DNP disorder emerged in mice overexpressing an IGF-1 inhibitory binding protein IGFBP5 [7] and the activation of Akt signaling, the crucial mediator of IGF-1 effects, are suppressed in neurons of DNP models both in vivo [26] and in vitro [27]. In our present study, high glucose-incubated neurons were employed as an in vitro model for studying the DNP, and upon high glucose insult, neurite sprouting was inhibited, neuronal viability was reduced, neuronal apoptosis was exacerbated, and the activation of Akt in DRG neurons was transiently inhibited as shown as the decreased pAkt level. Additionally, we found that the activation of S6K, a downstream kinase of Akt, whose activation is responsible for the synthesis of numerous structural proteins of neurons, was suppressed as well. Exogenous IGF-1 supplement partially reversed the alterations of neurite outgrowth, cell viability, neuronal apoptosis triggered by high glucose stimulation, and restored Akt and S6K phosphorylation, which was suppressed by pretreatment with LY294002. Both pAkt and pS6K levels were not significantly affected in the mannitol treated group, indicating that the possibly toxic effect of high osmotic pressure was beneath notice. These data indicate that the impairment of either Akt or S6K phosphorylation is related to the neurotoxic effect of high concentration glucose, and suggest that the mechanism underlying the neuroprotective action of IGF-1 against high glucose insult is based on the repair of the PI3K/Akt/ S6K signaling pathway.

The nervous system is susceptible to oxidative stress featured by overproduction of ROS such as superoxides, hydrogen peroxide, and nitric oxide, and high glucose exposure triggers abnormally elevated intracellular ROS level in neurons, which contributes to the pathogenesis and progression of DNP [3,28]. Here, in parallel with previous reports, we detected the elevated level of ROS inside neurons with high concentration glucose incubation, and this toxic effect could be rescue by administrating exogenous IGF-1. Several previous studies have shown the protective effects of IGF-1 against glucose toxicity of DRG neurons and other types of neurons by activating different signaling pathways. In high glucose treated DRG neurons, IGF-1 prevented neuronal apoptosis by regulating cyclic AMP response element binding protein (CREB), glycogen synthase kinase-3beta (GSK-3beta), and forkhead (FKHR) [29] (Leinninger et al, 2004) or by preventing mitochondrial accumulation of Bim and Bax [30] (Leinninger et al, 2006). IGF-1 prevented high glucose-induced apoptosis and neurite retraction in cultured superior cervical ganglion sympathetic neurons [31] (Russell and Feldman, 1999). IGF-1 also has ameliorating effects on apoptosis of human neuroblastoma cells with glucose deprivation [32] (Russo et al, 2004). IGF-1 significantly lowered intracellular ROS, which was accompanied by IGF-1-mediated FOXO3a nuclear export and decrease in its transcriptional activity in differentiated PC12 cells and rat cortical neurons with high glucose [33] (Wilk et al, 2011). Though it has been reported that increased intracellular ROS stimulates Akt signaling activation [34,35], in this study, PI3K/Akt/ S6K signaling inhibition with LY294002 reduced the antioxidative activity of exogenous IGF-1, indicating that there exists a negative feedback between PI3K/Akt/S6K signaling activation and intracellular ROS production.

Currently, ATF3, a transcription factor containing the basic region-leucine zipper (bZip) DNA binding domain, is usually used as a reliable marker for injury, since when exposed to stress signals, its expression is rapidly upregulated [36], and the elevated expression of ATF3 in DRGs has been reported to be closely related to the onset of neuropathic pain [37, 38, 39]. In our present study, ATF3 expression upregulation triggered by high glucose insult was partially blocked by pretreatment with exogenous IGF-1. The inhibitory actions of IGF-1 on ATF3 expression were also related to PI3K/Akt/S6K signaling, which was reflected by pretreatment LY294002 suppressed the effects produced by IGF-1. There was no significant alteration in ATF3 expression in mannitol treated neurons suggested the osmotic pressure induced by the corresponding concentration of mannitol or glucose did not affect the ATF3 expression. The toxic effects produced by high concentration glucose were related to the metabolism process of glucose itself rather than osmotic pressure. Moreover, ATF3 expression in situ paralleled its mRNA and protein expression, which further confirmed the involvement of alteration of ATF3 expression in DNP. These data imply that the upregulated ATF3 in DRG neurons might be involved in the genesis of DNP, and exogenous IGF-1 could suppress ATF3 upregulation in the presence of high glucose through restoration of PI3K/Akt/S6K signaling pathway.

In conclusion, the data in the present study imply that IGF-1 has powerful neuroprotective effects on DRG neurons with neurotoxicity induced by high glucose challenge. IGF-1 could promote neurite elongation, increase neuronal viability, alleviate apoptosis, reduce intracellular ROS production, and inhibit ATF3 expression in high glucose stimulation environment via restoration of impaired PI3K/Akt/S6K signaling. These results might contribute to clarification of the mechanism underlying the beneficial action of IGF-1 on reducing high glucose-triggered toxic actions, and shed light on clinical research for application of exogenous IGF-1 on treatment of intractable DNP.

## Abbreviations

ara-C: cytosine arabinoside
ATF3: activating transcription factor 3
CREB: cyclic AMP response element binding protein
DCFH-DA: 2′,7′-dichlorodihydrofluorescein diacetate
DNP: diabetic neuropathy
DRG: dorsal root ganglion
DSPN: distal symmetrical polyneuropathy
GAPDH: glyceraldehyde 3-phosphate dehydrogenase
IGF-1: insulin-like growth factor-1
MAP2: microtubule-associated protein 2
PBS: phosphate buffer saline
PCR: polymerase chain reaction
PI3K: phosphatidylinositol 3-kinase
ROS: reactive oxygen species
